# Predicting Unreported Micronutrients From Food Labels: Machine Learning Approach

**DOI:** 10.2196/45332

**Published:** 2023-04-12

**Authors:** Rouzbeh Razavi, Guisen Xue

**Affiliations:** 1 Department of Management and Information Systems Kent State University Kent, OH United States

**Keywords:** micronutrient deficiencies, micronutrient, food label, food, nutrition, nutrient, diet, machine learning, algorithm, predict, predictive model, nutrition mobile applications, mobile app, health app, mHealth, mobile health

## Abstract

**Background:**

Micronutrient deficiencies represent a major global health issue, with over 2 billion individuals experiencing deficiencies in essential vitamins and minerals. Food labels provide consumers with information regarding the nutritional content of food items and have been identified as a potential tool for improving diets. However, due to governmental regulations and the physical limitations of the labels, food labels often lack comprehensive information about the vitamins and minerals present in foods. As a result, information about most of the micronutrients is absent from existing food labels.

**Objective:**

This paper aims to examine the possibility of using machine learning algorithms to predict unreported micronutrients such as vitamin A (retinol), vitamin C, vitamin B1 (thiamin), vitamin B2 (riboflavin), vitamin B3 (niacin), vitamin B6, vitamin B12, vitamin E (alpha-tocopherol), vitamin K, and minerals such as magnesium, zinc, phosphorus, selenium, manganese, and copper from nutrition information provided on existing food labels. If unreported micronutrients can be predicted with acceptable accuracies from existing food labels using machine learning predictive models, such models can be integrated into mobile apps to provide consumers with additional micronutrient information about foods and help them make more informed diet decisions.

**Methods:**

Data from the Food and Nutrient Database for Dietary Studies (FNDDS) data set, representing a total of 5624 foods, were used to train a diverse set of machine learning classification and regression algorithms to predict unreported vitamins and minerals from existing food label data. For each model, hyperparameters were adjusted, and the models were evaluated using repeated cross-validation to ensure that the reported results were not subject to overfitting.

**Results:**

According to the results, while predicting the exact quantity of vitamins and minerals is shown to be challenging, with regression *R*^2^ varying in a wide range from 0.28 (for magnesium) to 0.92 (for manganese), the classification models can accurately predict the category (“low,” “medium,” or “high”) level of all minerals and vitamins with accuracies exceeding 0.80. The highest classification accuracies for specific micronutrients are achieved for vitamin B12 (0.94) and phosphorus (0.94), while the lowest are for vitamin E (0.81) and selenium (0.83).

**Conclusions:**

This study demonstrates the feasibility of predicting unreported micronutrients from existing food labels using machine learning algorithms. The results show that the approach has the potential to significantly improve consumer knowledge about the micronutrient content of the foods they consume. Integrating these predictive models into mobile apps can enhance their accessibility and engagement with consumers. The implications of this research for public health are noteworthy, underscoring the potential of technology to augment consumers’ understanding of the micronutrient content of their diets while also facilitating the tracking of food intake and providing personalized recommendations based on the micronutrient content and individual preferences.

## Introduction

### Background and Motivation

According to the World Health Organization (WHO), more than 2 billion people globally experience deficiencies in micronutrients such as vitamins and minerals [1]. Pregnant and nursing mothers and young children are particularly vulnerable to micronutrient deficiencies [2]. Insufficient intake of micronutrients has also been linked to disability-adjusted life years, a measure of time lost due to premature mortality and nonfatal health loss, and to various age-related disorders such as cardiovascular diseases and Alzheimer disease [3]. Food labels can be an effective tool for improving diets as they provide consumers with information to make healthier choices and decisions at the point of purchase and consumption [4-6]. In addition, increased consumer attention to nutrition labels has motivated food manufacturers to produce healthier products [7]. A systematic review of studies finds that reading nutrition labels is associated with healthier diets in adults [8].

Despite their potential benefits, food labels have limitations. In particular, including too much nutritional information on labels may be impractical due to size and ergonomic constraints [9]. Additionally, studies have found that clutter and information overload can overwhelm consumers, leading to the underutilization of labels [10]. As a result, food labels aim to convey only the most crucial nutritional information and are subject to revisions and amendments as people’s eating habits and the body of scientific knowledge regarding the impact of nutrition on health evolves. For example, the US Nutrition Facts label (NFL) first appeared in 1994 and was revised in 2016. The revision required US food manufacturers to report potassium and vitamin D and made the reporting of vitamins A and C voluntary. Data from the National Health and Nutrition Examination Survey (NHANES 2007-2011) suggests that about 92.4% of US adults fall short of the estimated average requirement for consuming vitamin D [11]. However, the decision to remove vitamins A and C from the list of micronutrients required to appear on food labels was not made because Americans received sufficient amounts of these vitamins. In fact, NHANES 2007-2011 indicated that 51.0% and 42.9% of adult Americans were not meeting dietary requirements for consuming vitamins A and C, respectively, even after accounting for these vitamins in fortified foods [11]. There are also many other unreported micronutrients that are underconsumed by the public. For example, according to NHANES 2007-2011, the vitamin E intake is below EAR for 93.9% of US adults, and magnesium and vitamin K deficiencies are observed among 60.9% and 71.1% of US adults, respectively. Micronutrient deficiencies are significantly more common in low-income countries [1].

It is well established that many nutrients exhibit synergistic interactions, whereby a deficiency in 1 nutrient may exacerbate or manifest as a deficiency in another, and vice versa. Furthermore, there are strong correlations between the presence of certain nutrients in various foods. For example, vitamin E, a fat-soluble antioxidant, tends to be positively correlated with the fat content of foods. Similarly, there is a strong positive correlation between dietary phosphorus and dietary calcium in various foods [12]. This study aims to explore the potential for estimating unreported micronutrients in foods using the nutrition information provided on labels. This is done by training a wide range of predictive machine learning algorithms using nutrition data from over 5000 foods. If successful, this could offer numerous benefits and options for consumers. For example, mobile apps could be developed to allow consumers to scan food labels and receive additional interactive information on the micronutrients of their interest that is not reported on labels.

The micronutrients examined in this study are those that are not currently reported on the NFL, including vitamin A (retinol), vitamin C, vitamin B1 (thiamin), vitamin B2 (riboflavin), vitamin B3 (niacin), vitamin B6, vitamin B12, vitamin E (alpha-tocopherol), vitamin K, and minerals such as magnesium, zinc, phosphorus, selenium, manganese, and copper. These micronutrients play vital roles in overall well-being, and their deficiency can lead to various health issues, as outlined in Multimedia Appendix 1.

### Related Studies

In recent years, the use of machine learning has opened up new avenues for predicting and estimating food attributes, including nutrient content [13]. Numerous studies have attempted to estimate food nutrients based on images of foods, which can help estimate food calories, analyze people’s eating habits, and make food recommendations. For example, the research in [14] introduces a new deep learning architecture specifically designed to handle the vertical food traits common to a large number of categories. Similarly, the authors in [15] propose a method for food image recognition using a fine-tuned deep convolutional neural network pretrained with ImageNet data. In [16], the authors propose a mobile-based dietary assessment system that can record real time images of the meal and analyze it for nutritional content, ultimately leading to improved dietary habits and a healthier lifestyle. The proposed system uses different machine learning models for accurate food identification and extracts semantically related words from a huge corpus of text collected over the internet to estimate food attributes and ingredients. The systematic review in [17] provides a comprehensive review of the application of deep learning in the food domain, including food classification, quality detection, and calorie estimation.

In addition to research related to food image analysis, machine learning has been used to predict food nutrient content in several studies. In 1 study, multiple machine learning algorithms were used to predict the carbohydrates, protein, and sodium content of foods [13]. Similarly, the fiber content in packaged foods was estimated using a k-nearest neighbor algorithm in [18], and added sugar content was predicted using the same method in [19]. While these studies provide valuable insights into the predictability of certain nutrients based on other nutrition information, it should be noted that the predicted nutrition attributes are typically those already reported on food package labels. In contrast, no previous study has focused on predicting those micronutrients that are not reported on food packages but are associated with deficiencies in a large portion of the population.

**Figure 1:**
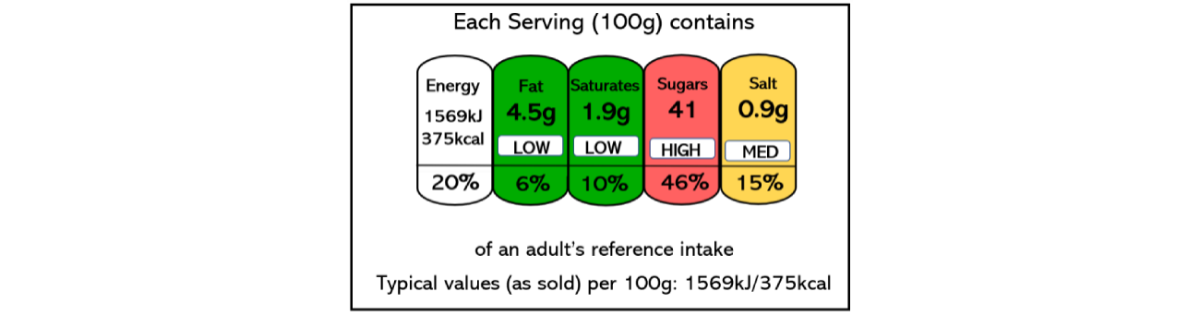
An example of Multiple Traffic Light (MTL) nutrition label developed by Food Standards Agency (FSA).

The prediction of unreported micronutrients has the potential to assist consumers in making informed choices about their dietary habits. However, it is crucial to present this information in an easily interpretable format for consumers. Franklin et al [20] found that a significant portion of the population faces difficulty understanding current food labels. To address this issue and avoid information overload, various label designs have been proposed. Among these designs is the Multiple Traffic Light system, developed by the UK Food Standards Agency, which uses traffic light colors and optional descriptors such as “low,” “medium,” and “high” to indicate reference intake levels for fat, saturated fat, sugars, and salt (as illustrated in Figure 1).

In comparison to traditional nutrition labels, mobile apps have the potential to significantly enhance consumers’ experiences and engagement in the context of predicting unreported micronutrients. These apps can be customized to meet individual consumers’ needs and preferences, such as allowing them to select specific micronutrients of interest, altering the way in which information is presented, choosing the level of detail, and comparing similar food products [21]. In addition, mobile apps may facilitate tracking daily or weekly nutrient intake and alerting users to any deficiencies. A systematic review by Coughlin et al [21] found that nutrition and diet-related mobile apps may be effective in promoting healthy behaviors and facilitating positive behavior change in various countries. Existing mobile apps such as Cronometer and Nutrients provide some micronutrient information for certain foods. However, these apps have limited databases. By predicting unreported micronutrients from existing food label information, such apps can provide micronutrient information about a much larger array of foods. Moreover, such mobile app integration will also allow apps to track users’ micronutrient intake over time and suggest foods to ensure that users consume sufficient amounts of various micronutrients. For example, the study in [22] presents a machine learning approach to recipe development based on nutrients in food, which can improve the nutritional quality of diets and prevent chronic diseases.

## Methods

### Nutrition Data

This study relies on nutritional data obtained from the Food and Nutrient Database for Dietary Studies (FNDDS), which is made available by the US Department of Agriculture (USDA). The FNDDS database is comprised of food intake information obtained from the What We Eat In America survey, which is conducted in collaboration between the USDA and the US Department of Health and Human Services (DHHS). The survey involves 2 interviews, the first conducted in person and the second via telephone within a time frame of 3 to 10 days, during which participants are asked to recall their dietary intake for 2 consecutive 24-hour periods. While the USDA is responsible for the dietary data collection methods, database maintenance, and data processing and evaluation, DHHS oversees sample design and data collection. Widely used in nutrition research, dietary assessment, and policy development, the FNDDS database is regarded as a reliable data set in the field [23].

We used nutrition data from 5624 foods included in the latest release of the FNDDS data set (ie, FNDDS 2019-2020) for our study, and no missing values were observed in the data set. The USDA’s Food Patterns Equivalents Database provides information about the food groups targeted in dietary guidance, and each food item in the FNDDS data set is associated with 1 of the 37 food groups defined in Food Patterns Equivalents Database. The independent variables in our research are the nutrition data provided on the current NFL, including calories, total fat, saturated fat, trans fat, cholesterol, sodium, total carbohydrates, dietary fiber, total sugars, protein, vitamin D, calcium, iron, and potassium. The mean, SD, and important percentiles of these attributes are presented in Table 1.

Furthermore, Table 2 presents the mean, SD, and important percentiles of the unreported micronutrients that serve as dependent variables in this study, all of which were derived from the FNDDS data set.

**Figure 2:**
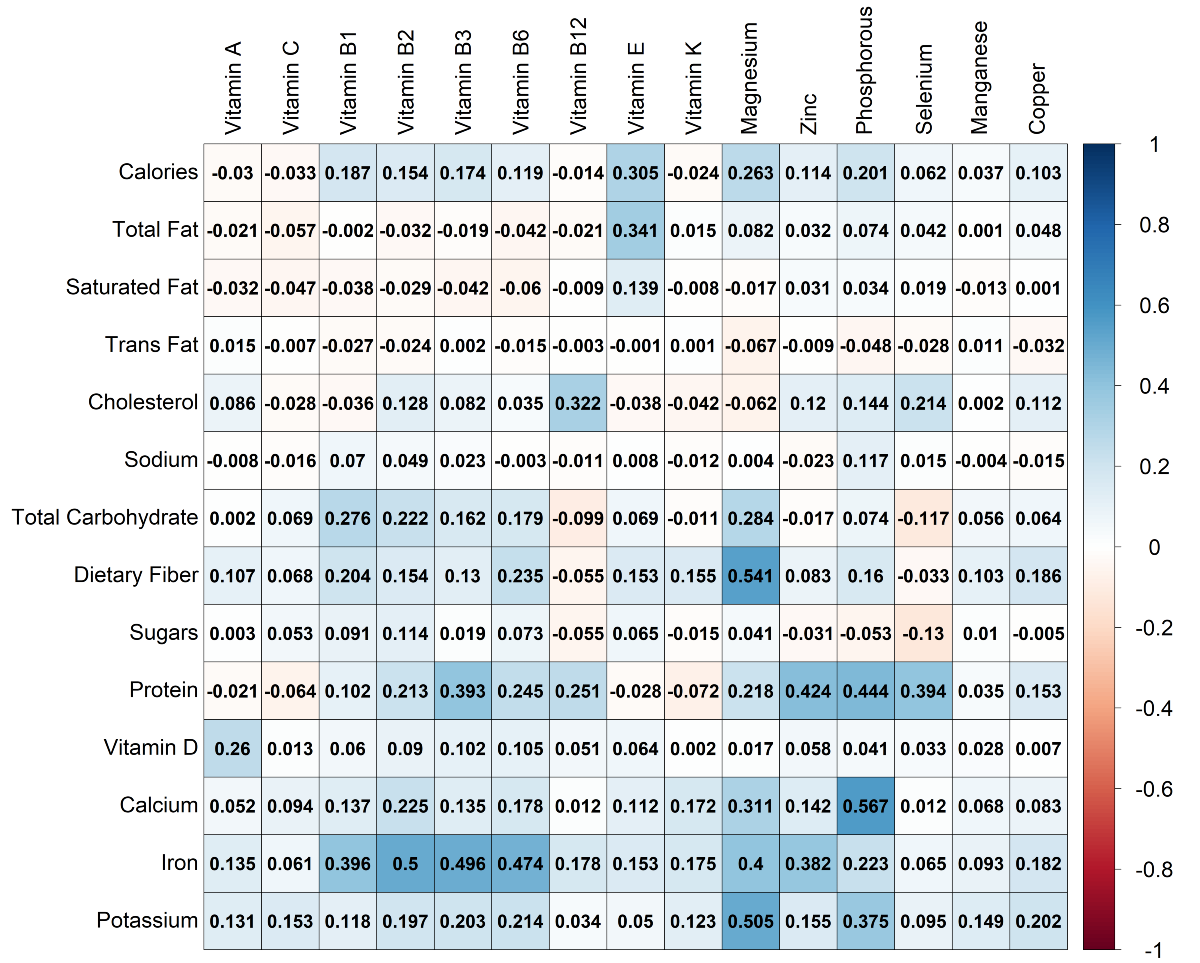
Correlation coefficients between independent (see Table 1) and dependent (see Table 2) nutrition attributes in this study.

Figure 2 illustrates the correlation coefficients between the nutrition variables shown in Table 1 (ie, independent variables in this study) and the micronutrients being predicted (presented in Table 2). Strong positive correlations between certain nutrients can be observed. While the co-occurrence of some of these nutrients, such as magnesium and dietary fiber [24], calcium and phosphorus [25], and zinc and protein [26], has been documented in the literature, it remains unclear whether these correlations are sufficient to accurately approximate unreported micronutrients. To address this question, a range of machine learning regression and classification models are used. Regression models are used to predict the quantities of micronutrients, while classification models aim to identify the category (ie, “low,” “medium,” or “high”) of micronutrients for each food, as will be discussed later.

**Table 1:** The mean, SD, and important percentiles of nutrition attributes (per 100 g of foods) listed on the Nutrition Facts label used in this study.

			Value, mean (SD)		Percentile												
					Min		25th		33rd		50th (median)		66th		75th		Max
Calories (kcal)			226.3 (169.86)		0.00		91.0		123.00		191.0		271.0		337.0		902.0
**Total fat (g)**			10.55 (15.81)		0.00		0.95		2.00		5.13		9.98		13.72		100.0
	Saturated fat (g)	3.43 (6.42)		0.00		0.14		0.41		1.43		2.94		4.17		95.61	
	Trans fat (g)	0.26 (1.45)		0.00		0.00		0.00		0.003		0.08		0.16		24.35	
Cholesterol (mg)			38.72 (117.35)		0.00		0.00		0.00		2.00		40.0		65.0		3100
Sodium (mg)			306.4 (939.22)		0.00		36.0		55.0		84.0		281.0		396.0		6681.76
**Total carbohydrate (g)**			22.12 (27.26)		0.00		0.05		2.40		9.34		20.87		34.91		100.0
	Dietary fiber (g)	2.04 (4.26)		0.00		0.00		0.00		0.04		1.60		2.40		26.32	
	Total sugars (g)	6.75 (13.71)		0.00		0.00		0.00		0.49		3.00		6.05		99.80	
Protein (g)			11.35 (10.53)		0.00		2.38		3.69		8.02		14.80		19.88		76.99
Vitamin D (mcg)			0.36 (3.09)		0.00		0.00		0.00		0.00		0.00		0.02		21.86
Calcium (mcg)			73.32 (199.85)		0.00		8.00		11.00		19.00		39.00		63.00		2003
Iron (mg)			2.76 (5.66)		0.00		0.49		0.73		1.30		2.08		2.57		64.55
Potassium (mcg)			265 (371.4)		0.00		111.0		143.0		219.0		290.0		329		3535

**Table 2:** The mean, SD, and important percentiles of micronutrients (per 100 g) that are not listed on the Nutrition Facts label and are dependent variables in this study are derived from the Food and Nutrient Database for Dietary Studies (FNDDS) data set.

	Value, mean (SD)	Percentile						
		Min	25th	33rd	50th (median)	66th	75th	Max
Vitamin A (mcg)	203.12 (469.12)	0.00	0.00	1.80	6.00	30.43	62.2	10,851.18
Vitamin B1 (mg)	0.21 (0.51)	0.00	0.03	0.04	0.07	0.13	0.22	23.37
Vitamin B2 (mg)	0.23 (0.44)	0.00	0.04	0.07	0.15	0.21	0.26	4.38
Vitamin B3 (mg)	3.40 (4.76)	0.00	0.37	0.68	2.11	4.01	5.06	45.24
Vitamin B6 (mg)	0.26 (0.46)	0.00	0.03	0.06	0.12	0.24	0.35	6.13
Vitamin B12 (mcg)	1.20 (4.27)	0.00	0.00	0.03	0.07	0.54	1.27	54.68
Vitamin C (mg)	8.36 (46.70)	0.00	0.00	0.01	0.29	0.90	2.50	240.92
Vitamin E (mg)	0.88 (2.81)	0.00	0.00	0.09	0.19	0.29	0.46	40.02
Vitamin K (mcg)	9.30 (22.15)	0.00	0.00	0.30	1.184	1.90	3.40	183.90
Magnesium (mg)	32.26 (45.80)	0.00	10.00	13.00	20.00	25.00	29.00	290.38
Zinc (mg)	1.94 (2.33)	0.00	0.22	0.48	0.80	1.70	2.66	12.50
Phosphorus (mg)	153.8 (202.20)	0.00	35.0	61.00	129.0	191.0	215.0	774.38
Selenium (mcg)	12.46 (27.07)	0.00	0.10	0.60	4.00	14.0	21.30	172.81
Manganese (mg)	0.49 (4.30)	0.00	0.00	0.02	0.05	0.12	0.30	52.26
Copper (mg)	0.16 (0.54)	0.00	0.02	0.04	0.07	0.10	0.14	5.12

### Machine Learning Algorithms

#### Classification and Regression Models

In addition to linear models, such as ordinary least squares (OLS) regression or logistic regression classification models, a range of machine learning regression and classification algorithms are deployed to capture the nonlinear and complex relationships between the dependent and independent variables in this study. The no free lunch theorem (NFLT) in machine learning [27] states that no one algorithm is optimal for every classification or regression problem, and therefore, a variety of algorithms are used to ensure that the reported predictive power of input attributes is not limited by a particular algorithm. A brief description of the algorithms used in this study is provided below. Those interested in further information about these algorithms are encouraged to refer to [28].

#### Decision Trees

Decision trees are a widely used algorithm that is popular due to their low computational complexity and transparency, making their output easy to interpret. These trees are constructed through a series of if-then rules based on the value of input features. Classification and regression trees are a widely deployed implementation of decision trees, and through the concept of ensemble learning, several individual decision trees can be combined to form random forests (RFs). The RF algorithm is based on the construction of a multitude of decision trees at training time and outputting the class that is the mode of the classes (classification) or mean prediction (regression) of the individual trees. This technique provides more robust and accurate predictions. Gradient boosting machines (GBMs) are also a popular tree-based algorithm that uses the concept of boosting, in which an iterative approach is taken to construct an ensemble model with each individual tree aimed at improving overall performance. GBMs differ from RFs by modifying the weights of the training data at each iteration, allowing for a greater emphasis on incorrectly classified data points in subsequent iterations and thus improving the overall accuracy of the model.

#### K-Nearest Neighbors

The k-nearest neighbors (KNN) is a simple yet powerful algorithm that makes predictions for any record by finding the k most similar data points in the training data set. KNN uses a distance metric, such as Euclidean distance, to measure the similarity between a test sample and the training data set. The algorithm then identifies the k closest neighbors to the test sample based on the distance metric and assigns the class of the majority of the nearest neighbors to the test sample. This algorithm is nonparametric and does not require a training phase, making it easy to implement and apply to various data sets.

#### Neural Networks

Neural networks are a complex and powerful class of machine learning algorithms that are based on the structure and function of the human brain. They consist of several simple, highly interconnected processing elements known as artificial neurons that are structured in layers and connected through weighted links. The aim of this algorithm is to simulate the behavior of the human brain, with neurons activated by other neurons to which they are connected and, once triggered, generate output that can trigger additionally connected neurons. The neural network is composed of an input layer, one or more hidden layers, and an output layer. Neural networks can be used for both classification and regression tasks, and their power lies in their ability to capture complex and nonlinear relationships between the input features and the output variable.

#### Support Vector Machines

Support vector machines (SVMs) aim to find optimal decision planes that serve as decision boundaries. For this, SVM uses the concept of kernel methods. In this method, the input data is mapped onto a high-dimensional feature space using a set of functions known as kernels, where decision boundaries can be defined. Linear kernels and radial basis function (RBF) kernels are 2 widely used SVM kernel functions. SVMs are particularly effective in high-dimensional spaces, where the number of features is large compared to the number of samples. SVMs can be used for both regression and classification tasks and can handle complex data sets with nonlinear boundaries.

It should be noted that these algorithms are parameterized, and the choice of their tuning parameters, also referred to as hyperparameters, can significantly impact their performance [28,29]. Table 3 lists the machine learning algorithms deployed in this study along with their corresponding hyperparameters.

As shown in Table 3, the study deploys both classification and regression models to predict the micronutrient contents of foods. While regression models attempt to predict the exact nutrient quantities, it should be noted that most consumers may not need the exact numeric quantities of food micronutrients. This is due to the wide range of daily recommended values for micronutrients, which can range from micrograms to milligrams. As a result, interpreting the numeric quantities of micronutrients can be challenging for many consumers [20]. To address this issue, food authorities around the globe have developed simplified food labels, such as the Multiple Traffic Light designs by the UK Food Standards Agency (see Figure 1). Similarly, in this study, 3 categories of low, medium, and high are also considered for each micronutrient based on the percentiles of their distribution (see Table 2). The “low” category represents values that are below the 33rd percentile; the “medium” category refers to values between the 33rd and 66th percentiles; and the “high” category refers to values above the 66th percentile. These labels can then be used to represent the various micronutrient contents. From a modeling perspective, these will be 3-level classification tasks and are examined using the classification algorithms shown in Table 2. In order to avoid overfitting, the data was divided into 2 mutually exclusive subsets: the training set (80%) and the test set (20%) [28,29]. A methodology introduced in [29] was used for this purpose, with the training and test sets being determined at random but possessing similar distributions of the dependent variable in both sets. The optimal values of the hyperparameters were subsequently determined through an adaptive search algorithm proposed by Kuhn and Johnson [29] and 10-fold cross-validation using only the training set. In k-fold cross-validation, data is randomly divided into k equal partitions, each referred to as a fold, with k-1 folds being used to construct a model and the remaining fold being used for performance evaluation. This process is repeated k times, with a different fold chosen for evaluation each time. Once the optimal values of the hyperparameters are determined, the entire training set is used to train a model, the performance of which is then evaluated on the test set. This approach ensures that the test set samples are not used during the model training or hyperparameter optimization steps, thereby minimizing the risk of overfitting [28].To ensure that performance evaluations are not specific to any one random test set (which could potentially consist only of easily predictable samples, for example), the above procedure is replicated 10 times, each time using new random training and test sets, and the performance measures are averaged [29]. The evaluation of models’ performance based on 10 independent, random, and completely out-of-sample test sets is deemed critical in ensuring that the results reported in this study are not subject to overfitting, a risk when applying advanced machine learning algorithms [28,29].

**Table 3:** List of machine learning algorithms deployed in this study along with their hyperparameters.

Algorithm name	Acronym	Application	Hyperparameters
Classification and regression trees	CART	Classification and regression	Complexity parameter, *cp*
Gradient boosting machines	GBM	Classification and regression	No. of trees, *n.trees* Depth, *interaction.depth* Shrinkage parameter, *shrinkage*
K-nearest neighbors	KNN	Classification and regression	No. of neighbors, *k*
Logistic regression	LR	Classification	N/A^a^
Neural networks	NN	Classification and regression	Weight decay, *decay* No. of hidden layer units, *size*
Ordinary least squares	OLS	Regression	N/A
Random forests	RF	Classification and regression	No. of variables available for splitting, *mtry*
Support vector machines–linear kernel	SVM-L	Classification and regression	Cost parameter, *c*
Support vector machines–radial basis function kernel	SVM-RBF	Classification and regression	Cost parameter, *c* RBF kernel parameter, *sigma*

^a^N/A: not applicable.

## Results

### Regression Models

**Figure 3:**
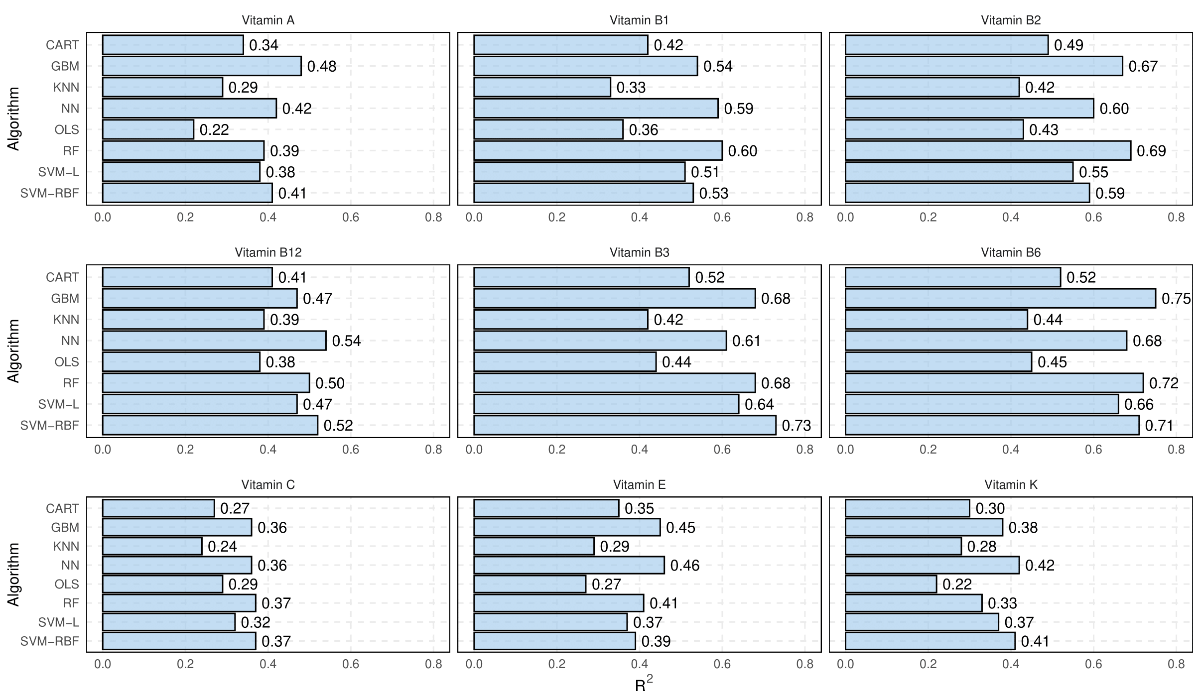
R2 of different regression models for predicting unreported vitamins using other nutrition information presented on Nutrition Facts Label. CART: classification and regression trees; GBM: gradient boosting machines; KNN: k-nearest neighbors; NN: neural networks; OLS: ordinary least squares; RF: random forests; SVM-L: support vector machines-linear kernels; SVM-RBF: support vector machines-radial basis function.

Figure 3 shows the *R*^2^ of different regression models for predicting unreported vitamins using the nutrition information provided by the NFL. It is found that vitamin B6 could be predicted with the highest accuracy, with an *R*^2^ of 0.75 for the best model, namely, GBM. In contrast, the prediction of vitamin C is found to be the most challenging, with an *R*^2^ of 0.37 for the best model, namely, RFs. The results also demonstrate the superiority of most machine learning algorithms over the linear regression model (OLS), as the former is able to capture complex and nonlinear relationships that may exist between the predictors and dependent variables.

**Figure 4:**
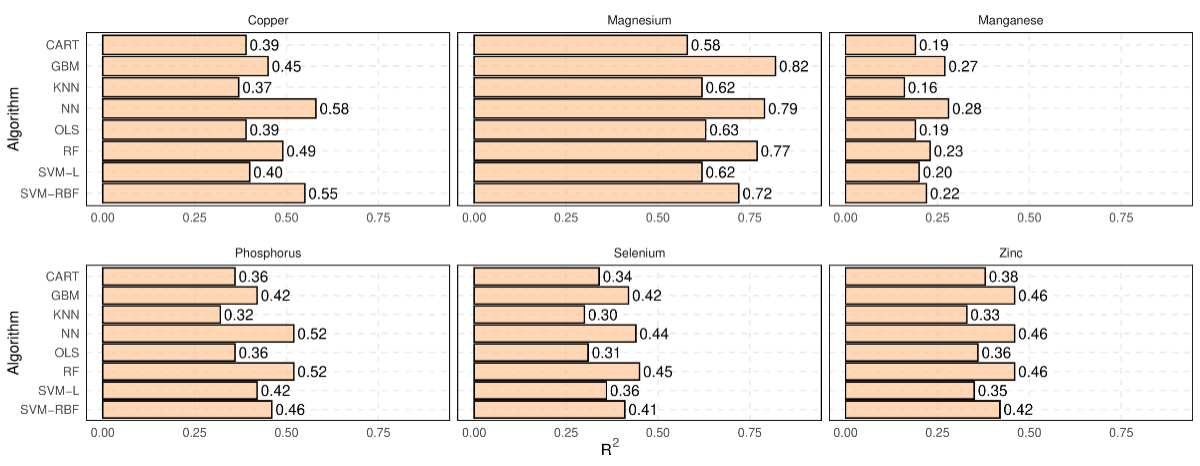
R2 of different regression models for predicting unreported minerals using other nutrition information presented on Nutrition Facts Label. CART: classification and regression trees; GBM: gradient boosting machines; KNN: k-nearest neighbors; NN: neural networks; OLS: ordinary least squares; RF: random forests; SVM-L: support vector machines-linear kernels; SVM-RBF: support vector machines-radial basis function.

Similarly, Figure 4 shows the *R*^2^ of various regression models for estimating unreported minerals. As shown, magnesium can be estimated with the highest accuracy (*R*^2^=0.82 for the best model, ie, GBM), while the accuracy of predicting manganese is the lowest compared to other minerals (*R*^2^=0.28 for the best model, ie, NN). Again, the result confirms that most machine learning algorithms outperform OLS.

The results from Figures 3 and 4 show that while the use of machine learning models can explain a significant portion of the variability in unreported micronutrients based on the nutrition information provided on food labels, the accuracy of such models in predicting the exact value of these micronutrients is far from perfect. However, as discussed, the exact numeric value of a micronutrient may not always be necessary. The next section presents the results of the classification models.

### Classification Models

**Figure 5:**
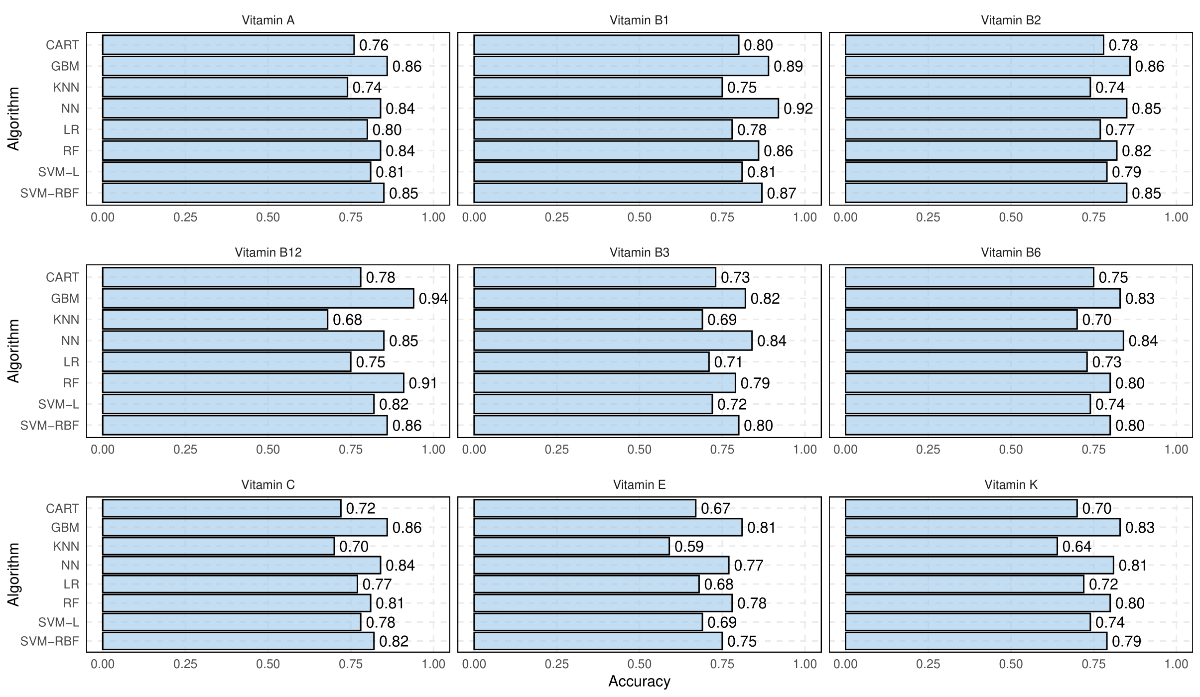
Accuracy of different classification models for predicting the category of unreported vitamins using other nutrition information presented on Nutrition Facts Label. CART: classification and regression trees; GBM: gradient boosting machines; KNN: k-nearest neighbors; LR: logistic regression; NN: neural networks; OLS: ordinary least squares; RF: random forests; SVM-L: support vector machines-linear kernels; SVM-RBF: support vector machines-radial basis function.

The accuracy of various classification models for predicting the category (ie, “low,” “medium,” or “high”) of unreported vitamins using the nutrition information provided on NFL is shown in Figure 5. In this context, classification accuracy is defined as the proportion of correctly classified records. It can be seen that reasonable accuracy is achievable for predicting the categories of most vitamins. When comparing different vitamins, the results suggest that the accuracy is particularly high (>0.90) for the best machine learning models when predicting the presence of vitamins B1 and B12. In general, nonlinear models, such as GBM, SVM, RF, and NN, are shown to outperform linear logistic regression models and simple decision trees (classification and regression tree).

Further examination of the misclassified records in Figure 5 reveals 2 notable characteristics. First, a significant percentage of misclassified records are misclassified between the “low” and “medium” categories or between the “medium” and “high” categories, with a negligible percentage between the “low” and “high” categories. For example, considering the best classification model for vitamin A (ie, GBM), 13.82% of foods are misclassified. This includes 3.42% of foods in the “low” vitamin A category being classified as “medium,” 2.98% of foods from the “medium” category being classified as “low,” 3.85% of foods from the “medium” class being classified as “high,” 3.35% of foods from the “high” class being classified as “medium,” and only 0.18% of foods from the “low” category being classified as “high,” and 0.04% of foods from the “high” category being misclassified as “low.” Second, an examination of the misclassified records reveals that most are borderline. For example, considering the GBM model for vitamin A prediction, the average percentile of “low” category records that are misclassified as “medium” is 30.78%, which is close to the classification boundary (ie, 33%). Similarly, the average percentile of “medium” category records that are misclassified as “low” and “high” is 38.56% and 60.24%, respectively. These observations suggest that not only is the rate of misclassification relatively small, but also that those records that are misclassified are not severely misleading.

**Figure 6:**
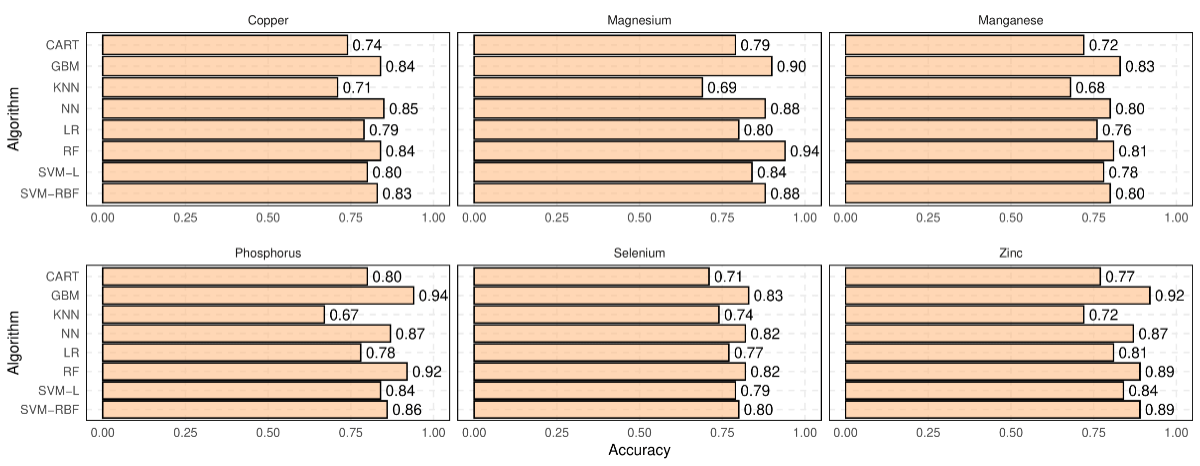
The accuracy of different classification models for predicting unreported minerals using other nutrition information presented on Nutrition Facts Label. CART: classification and regression trees; GBM: gradient boosting machines; KNN: k-nearest neighbors; LR: logistic regression; NN: neural networks; OLS: ordinary least squares; RF: random forests; SVM-L: support vector machines-linear kernels; SVM-RBF: support vector machines-radial basis function.

Similarly, Figure 6 shows the accuracy of different classification models for predicting the category (ie, “low,” “medium,” or “high”) of unreported vitamins using the nutrition information provided on the NFL. Again, we can see a reasonable performance of classification models, where the best models’ accuracy is higher than 90% for predicting magnesium, phosphorous, and zinc.

The results in Figure 6 demonstrate the reasonable performance of classification models in predicting the categories of unreported minerals, with the best models achieving accuracy higher than 0.90 for predicting magnesium, phosphorous, and zinc. In order to gain a deeper understanding of the role of each independent variable in predicting unreported micronutrients, Table 4 presents variable importance scores (VIS) for each predictor nutrition attribute when predicting various unreported vitamins. As described in [29], VIS is calculated by removing independent variables from a model and measuring the corresponding decrease in the model’s accuracy. The most important attribute is assigned a value of 100, with other attributes scaled proportionally.

The results in Table 4 align with the correlation results shown in Figure 2, with protein, iron, and potassium frequently appearing as highly predictive independent attributes when classifying foods based on their vitamin content. Similarly, Table 5 presents the VIS values for each nutrition attribute when predicting various unreported minerals, with the results again agreeing with the correlation values shown in Figure 2, where potassium, iron, calcium, and protein are among the top predictors.

**Table 4:** The variable importance score quantities for each of the reported nutrition attributes when predicting various unreported vitamins.

	Vitamin A	Vitamin B1	Vitamin B2	Vitamin B3	Vitamin B6	Vitamin B12	Vitamin C	Vitamin E	Vitamin K
Calories	60.49	22.39	20.49	19.33	23.08	9.05	75.33	74.91^a^	49.51
Total fat	52.71	19.4	18.66	15.34	19.29	7.71	62.48	100^a^	65.89
Saturated fat	52.26	21.31	22.47	17.81	24.27	12.04	49.95	69.39	58.60
Trans fat	37.76	32.90	28.26	14.52	25.19	13.90	47.11	54.81	33.28
Cholesterol	41.76	29.71	16.30	9.69	15.14	100^a^	40.42	46.07	36.40
Sodium	67.32	27.92	25.92	17.43	25.10	14.91	65.93	63.87	56.19
Total carbohydrate	82.20^a^	48.60	16.70	18.73	22.71	17.62	100^a^	70.74	49.43
Dietary fiber	38.09	13.55	13.02	12.3	15.27	7.61	36.79	36.72	100^a^
Total sugars	50.58	12.31	12.95	10.97	21.56	6.35	50.32	60.10	37.93
Protein	86.07^a^	47.08^a^	100^a^	100^a^	82.64^a^	39.68^a^	82.5^a^	72.12	59.97
Vitamin D	55.97	17.50	11.66	5.68	9.765	27.95	29.47	78.3	64.50
Calcium	100^a^	22.09	33.15	24.59	29.9	21.15	66.82	73.31	76.27^a^
Iron	71.11	100^a^	55.31^a^	76.92^a^	100^a^	33.7^a^	71.85	75.35^a^	72.01
Potassium	74.89	45.91^a^	40.32^a^	38.12^a^	51.68^a^	28.42	86.42^a^	68.9	79.01^a^

^a^The top 3 predictors.

**Table 5:** The variable importance score quantities for each of the reported nutrition attributes when predicting various unreported minerals.

	Copper	Magnesium	Manganese	Phosphorus	Selenium	Zinc
Calories	24.2	17.19	38.21	10.97	20.06	8.57
Total fat	22.73	13.44	33.45	7.14	18.95	6.97
Saturated fat	26.7	16.24	37.32	9.88	21.39	11.78
Trans fat	19.91	15.90	41.4	10.48	19.76	9.10
Cholesterol	33.71	34.19	65.78	8.30	44.09^a^	4.74
Sodium	30.97	16.53	50.42	12.49	28.02	9.89
Total carbohydrate	28.68	17.21	72.45^a^	8.12	31.02	11.73
Dietary fiber	20.43	66.84^a^	72.25	6.09	17.06	8.36
Total sugars	20.95	10.67	49.20	5.05	18.98	6.11
Protein	31.97	37.87^a^	52.52	100^a^	100^a^	100^a^
Vitamin D	11.49	6.683	22.60	3.22	8.85	4.50
Calcium	34.64^a^	24.86	61.97	95.74^a^	38.21	33.43
Iron	100^a^	29.31	83.56^a^	12.11	27.19	55.38^a^
Potassium	70.86^a^	100^a^	100^a^	37.83^a^	48.51^a^	37.76^a^

^a^The top 3 predictors.

## Discussion

### Principal Findings

The results presented in the previous section demonstrate the potential for machine learning models to estimate unreported micronutrients based on the nutrition information provided on food labels. The regression models presented in this study showed that vitamin B6 and magnesium were the micronutrients that could be predicted with the highest accuracy, while vitamin C and manganese were the most challenging micronutrients to predict accurately. Furthermore, the classification models were able to predict the categories of most vitamins and minerals with a high degree of accuracy, with nonlinear models outperforming linear models. The VISs for each predictor nutrition attribute were also calculated to gain a deeper understanding of the role of each independent variable in predicting unreported micronutrients. The results indicate that protein, iron, potassium, and calcium were among the top predictors for both vitamins and minerals.

The findings of this study demonstrate that classification models outperform regression models in predicting unreported micronutrients using nutrition information provided on food labels. It is important to note that regression models aim to make precise predictions about continuous dependent variables, which makes them more complex than classification models that predict categorical outcomes based on input features. Furthermore, the skewed distribution of dependent nutrition variables considered in the regression models, as presented in Table 2, can significantly hinder their prediction accuracy. The interpretation of exact numeric quantities of food micronutrients, however, can be challenging for many consumers due to the wide range of daily recommended values [20], making it unnecessary for most to require exact numeric quantities. On the other hand, classification models, which indicate micronutrient levels as “low,” “medium,” or “high,” provide easily interpretable insights for consumers. Mobile apps that implement classification models to predict unreported micronutrients can offer beneficial information to various user groups, especially those at risk of micronutrient deficiencies, including vegetarians, vegans [30], and older adults [3,31]. By scanning food labels, mobile apps can provide a simplified and engaging presentation of nutrient content and also offer features such as daily meal planning and water intake tracking [32,33]. Fortunately, machine learning algorithms are easy to deploy on mobile apps, and they do not require privacy-sensitive permissions to function. Furthermore, mobile apps that monitor users’ food intake can suggest foods based on their micronutrient content. Such features have been reported to be effective in increasing dietary diversity and alleviating micronutrient deficiency [34]. In general, these features can raise user awareness of their health and nutrition and, as a result, may lead to better dietary habits [35].

### Limitations and Future Studies

While this research offers valuable insights into the potential for estimating various unreported micronutrients from existing food labels, it has some limitations that should be considered. Most importantly, this study focused on American diets using the foods listed in the What We Eat In America database. However, given that diets differ between countries [36], further research using diet and nutrient data from other countries would be valuable. In addition to the potential for improved performance with larger training data sets, it is worth considering that certain machine learning algorithms may perform better at predicting specific micronutrients or food categories. Therefore, the development of ensemble machine learning models that combine the strengths of individual models may represent a promising avenue for further enhancing predictive accuracy and should be pursued in future research. Finally, a follow-up study demonstrating the integration of machine learning algorithms to predict unreported micronutrients would provide significant practical value.

### Conclusions

This study demonstrates the feasibility of predicting unreported micronutrients from food labels. By analyzing detailed nutrition data from over 5000 foods and applying a range of machine learning algorithms, the study finds that it is possible to predict the category (ie, “Low,” “Medium,” or “High”) of various vitamins and minerals with an accuracy of at least 0.80. According to the results of this study, the highest prediction accuracies for specific micronutrients were achieved for vitamin B12 (0.94) and phosphorus (0.94), while the lowest were for vitamin E (0.81) and selenium (0.83). These findings have important implications for helping consumers make more informed decisions about their nutrition and for improving the overall health of the population. By using mobile apps to present this information in an engaging and easy-to-interpret manner, it may be possible to encourage better nutrition decisions and address the significant prevalence of micronutrient deficiencies.

## Appendix

Multimedia Appendix 1 The key role of micronutrients considered in this study and the health issues associated with their deficiency.

## Abbreviations

DHHS: Department of Health and Human Services
FNDDS: Food and Nutrient Database for Dietary Studies
GBM: gradient boosting machines
KNN: k-nearest neighbors
LR: logistic regression
NFL: Nutrition Facts labels
NHANES: National Health and Nutrition Examination Survey
OLS: ordinary least squares
RBF: radial basis function
RF: random forest
SVM: support vector machine
USDA: US Department of Agriculture
VIS: variable importance score
WHO: World Health Organization

## Data Availability

This study used the Food and Nutrient Database for Dietary Studies (FNDDS) 2019-2020 data set, which is made available by the US Department of Agriculture (USDA) and can be obtained from [37].
