# Supplementary material for: Predicting Unreported Micronutrients From Food Labels: Machine Learning Approach
Source: J Med Internet Res. 2023 Apr 12;25:e45332. doi: 10.2196/45332 (PMC10134025; doi:10.2196/45332)
Supplement: Multimedia Appendix 1 [file jmir_v25i1e45332_app1.docx]

## Multimedia Appendix A

The key role of micronutrients considered in this study and the health issues associated with their deficiency.

|  | Role and Health Issues Associated with Deficiency |
| --- | --- |
|  |  |
| Vitamin A | Essential for supporting a healthy immune system and good vision. Deficiency can lead to night blindness (nyctalopia) and keratomalacia, which can cause blindness if left untreated. |
| Vitamin B1 | Necessary for the growth, development, and function of cells, as well as for generating energy from carbohydrates. Deficiency can cause weight loss, emotional disorders, decreased sensory sensitivity, limb pain and weakness, and irregular heartbeat. |
| Vitamin B2 | Involved in the growth of cells, energy production, and the breakdown of fats, steroids, and medications. Deficiency can cause a painful red tongue with sore throat, and can also impact the function of the nervous system. |
| Vitamin B3 | Helps to maintain the health of the nervous system, digestive system, and skin. Deficiency can cause pellagra, which is characterized by diarrhea, dermatitis, dementia, and death. |
| Vitamin B6 | Important for brain development and for maintaining the health of the nervous system and immune system. Deficiency can cause microcytic anemia, irregular electroencephalograms, dermatitis with cheilosis, glossitis, depression, and impaired immunological function. |
| Vitamin B12 | Plays a crucial role in red blood cell formation, cell metabolism, nerve function, and DNA production. Deficiency can cause anemia, neurological disorders, and digestive disorders. |
| Vitamin C | Helps to protect cells against the damaging effects of free radicals. Deficiency can cause weakness, weight loss, and general pains. |
| Vitamin E | Important for maintaining healthy vision, reproduction, and the health of the blood, brain, and skin. Deficiency can impact the function of the nervous system. |
| Vitamin K | Necessary for helping the blood to clot and preventing excessive bleeding. Deficiency can cause sensitivity to bruises, nosebleeds, and bleeding gums. |
| Magnesium | Important for regulating blood sugar levels, blood pressure, and the production of protein, bone, and DNA. Deficiency can cause high blood pressure, osteoporosis, and migraines. |
| Zinc | Crucial for DNA synthesis, cell development, protein synthesis, tissue repair, and immune system support. Deficiency can cause decreased appetite, slower growth than expected, and a weakened immune system. |
| Phosphorus | Required by all tissues and cells for their growth, maintenance, and repair. Deficiency, known as hypophosphatemia, can lead to appetite loss, anxiety, bone pain, stiff joints, fatigue, uneven breathing, irritability, numbness, weakness, and weight change. |
| Selenium | Plays a key role in the proper functioning of the thyroid gland and helps to make DNA and protect against cell damage. Deficiency has been linked to autoimmune thyroid conditions, cardiovascular disease, and infertility. |
| Manganese | Aids in the body's formation of bones, connective tissue, blood clotting factors, and sex hormones. Epilepsy, osteoporosis, diabetes and exocrine pancreatic insufficiency are linked to deficiency. |
| Copper | Maintains the health of immune system, neuron cells, and red blood cells in body. Deficiency can lead to low white blood cell count, irregular heartbeat, loss of skin pigment, hypothyroidism, anemia, low body temperature, bone fractures, and osteoporosis |
